# Influence of Reverse Osmosis Process in Different Operating Conditions on Phenolic Profile and Antioxidant Activity of Conventional and Ecological Cabernet Sauvignon Red Wine

**DOI:** 10.3390/membranes12010076

**Published:** 2022-01-08

**Authors:** Ivana Ivić, Mirela Kopjar, Ivana Buljeta, Dubravko Pichler, Josip Mesić, Anita Pichler

**Affiliations:** 1 Faculty of Food Technology Osijek, Josip Juraj Strossmayer University, F. Kuhača 18, 31000 Osijek, Croatia; iivic@ptfos.hr (I.I.); mirela.kopjar@ptfos.hr (M.K.); ivana.buljeta@ptfos.hr (I.B.); 2 Water Supply-Osijek, Poljski Put 1, 31000 Osijek, Croatia; dubravko.pichler@vodovod.com; 3 Polytechnic in Požega, Vukovarska 17, 34000 Požega, Croatia; jmesic@vup.hr

**Keywords:** phenolic compounds, antioxidant activity, conventional and ecological red wine, reverse osmosis, retention

## Abstract

Red wine polyphenols are responsible for its colour, astringency, and bitterness. They are known as strong antioxidants that protect the human body from the harmful effects of free radicals and prevent various diseases. Wine phenolics are influenced by viticulture methods and vinification techniques, and therefore, conventionally and ecologically produced wines of the same variety do not have the same phenolic profile. Ecological viticulture avoids the use of chemical adjuvants in vineyards in order to minimise their negative influence on the environment, wine, and human health. The phenolic profile and antioxidant activity of wine can also be influenced by additional treatments, such as concentration by reverse osmosis. The aim of this study was to investigate the influence of four different pressures (2.5, 3.5, 4.5, and 5.5 MPa) and two temperature regimes (with and without cooling) on the phenolic profile and antioxidant activity of conventional and ecological Cabernet Sauvignon red wine during concentration by reverse osmosis. The results showed that retention of individual phenolic compounds depended on the applied processing parameters, chemical composition of the initial wine, and chemical properties of a compound. Higher pressure and retentate cooling favoured the retention of total polyphenols, flavonoids, and monomeric anthocyanins, compared to the opposite conditions. The same trend was observed for antioxidant activity.

## 1. Introduction

It is generally known that moderate red wine consumption contributes to human health, reducing the risks of various cardiovascular, degenerative, and other chronic diseases due to the antioxidant activity of red wine. Red wine contains different types of phenolic compounds that are responsible for its colour, bitterness, and astringency, and they also act as antioxidants [1]. Antioxidants protect the human body from harmful effects of free radicals and oxidative stress that can cause various diseases. They also have antimicrobial and anti-inflammatory effects [2,3]. Phenolic compounds in wine include many different types of compounds that are divided into nonflavonoids and flavonoids. The group of flavonoids includes flavonols, anthocyanins, and tannins, and the nonflavonoids group includes stilbenes, and hydroxybenzoic and hydroxycinnamic acids. The phenolic content in wines can range from 1800 to 3000 mg/L [4]. It differs between wine varieties and depends on the viticulture and vinification techniques. Climate conditions, grape maturity at harvest day, soil characteristics, maceration time and temperature, fermentation, and ageing and storage conditions are the main factors influencing the wine phenolic profile. Each wine variety usually has a stable and constant profile of anthocyanins, although it can sometimes vary due to previously mentioned factors [5]. Most of the phenolic compounds are located in skins and seed of a red grape berry, and they are extracted during crushing and maceration into grape juice [6]. Further, changes in the phenolic profile and antioxidant activity of red wine can occur during fermentation and ageing, or during additional treatment of wine.

Additional wine treatment is sometimes necessary if the chemical composition of the wine does not meet the standards. For example, a poor vintage or inadequate viticulture or vinification procedures can result in lower content of phenolics and lower antioxidant activity than desired. In such cases, membrane filtration, especially reverse osmosis (RO), can be used for wine concentration; partial dealcoholisation; and correction of chemical composition, aroma, or phenolic profile [6,7,8,9,10]. Reverse osmosis has several advantages over other thermal concentration processes, such as high selectivity, low energy consumption, a low cost, operation at room temperatures, and no chemical requirements for sample preparation [11]. The RO process includes the use of a selective membrane and high-pressure application. The selective membrane can be made of different materials, but its pore size cannot exceed 1 nm, or 200 Da (they retain molecules with a molecular weight higher than 200 g/mol) [12]. The pressure range for a brackish water RO process usually ranges between 1.0 and 2.5 MPa; for a seawater RO process, from 4.0 to 8.0 MPa [13]; and in the wine industry, from 2.0 to 6.0 MPa, or higher if necessary [14,15]. When high pressure is applied, the membrane splits the initial feed on the fraction that is retained on the membrane (retentate or concentrate) and the fraction that passes through it (permeate). High pressure is necessary to overcome the osmotic pressure that is created on the membrane surface by molecules that are retained on it. Wine permeate usually contains small molecules, mostly water and ethanol, but also several small aroma compounds or organic acids [14]. One of the biggest disadvantages of the RO process is the limitation of the process due to concentration polarization and membrane fouling. Membrane fouling is a result of the accumulation of matter during the concentration process on the membrane surface or in the membrane pores, which creates a cake and blocks the membrane. This results in lower permeate flux, requiring membrane cleaning [16]. However, advantages of the RO process still stand out, making it applicable in various industries (beer, dairy, wine, and others). In the wine industry, it is mostly used for wine concentration and ethanol removal, but the RO process is often used for correction of wine phenolic content. The RO process can be used as the first step for the extraction of polyphenols that are further used as functional ingredients [17]. Lamont et al. [18] stated that alcohol removal from wine by reverse osmosis does not significantly change wines’ cardioprotective properties and antioxidant capacities.

In this study, the reverse osmosis process was used for the concentration of conventional and ecological red wine. The main difference between conventional and ecological wine is the absence of chemical additives (fertilizers, pesticides, etc.) during ecological grape and wine production. Chemical adjuvants have a negative impact on the environment, soil, and human health, and the main goal of ecological wine production is to minimise this negative impact [19,20]. Further, during the harvest of ecologically produced grapes, machinery is also avoided, and berries are collected by hand in order to minimise the mechanical damage to the berries [19]. In order to convert the vineyard from conventional to ecological viticulture, several years of special soil pre-treatment and clean water are required [21]. After the accreditation procedure, the vineyard can acquire a certificate that indicates a precise location and starting date of ecological viticulture [20,22].

The aim of this study was to investigate the influence of pressure (2.5, 3.5, 4.5, and 5.5 MPa) and temperature regime (with and without cooling) during the reverse osmosis process of conventional and ecological Cabernet Sauvignon red wine. In initial wines and obtained retentates, the total polyphenols, flavonoids, monomeric anthocyanins contents, individual phenolic compounds, antioxidant activity, and CIELab colour parameters were determined. The effect of wine type and different operating conditions on the above-mentioned parameters was monitored.

## 2. Materials and Methods

### 2.1. Chemicals and Standards

Chemicals and standards used in this study were obtained from: Sigma-Aldrich, St. Louis, MO, USA (Trolox, 2,2-diphenyl-1-picrylhydrazil (DPPH), 2,2-azinobis(3-ethylbenzothiazoline sulfonic acid) (ABTS), 2,4,6-tripyridyl-s-triazine (TPTZ), aluminium chloride, quercetin dihydrate, gallic acid monohydrate, and potassium persulfate); Sigma-Aldrich Chemie Gmbh, Steinheim, Germany (gallic acid, caffeic acid, (+)-catechin hydrate, (-)-epicatechin, rutin hydrate, and quercetin); Extrasynthese, Genay, France (malvidin-3-glucoside); Kemika, Zagreb, Croatia (Folin–Ciocalteu reagent, sodium nitrite, sodium carbonate, sodium hydroxide, potassium bisulphite, sodium acetate, potassium chloride, and hydrochloric acid); Gram-Mol, Zagreb, Croatia (sodium acetate trihydrate, ferric chloride hexahydrate, and ammonium acetate); Acros Organics, New Jersey, NJ, USA (copper(II) chloride); Merck, Darmstadt, Germany (HPLC grade methanol and neocuproine); Fluka, Buchs, Switzerland (HPLC-grade phosphoric acid).

### 2.2. Cabernet Sauvignon Red Wine

In this study, conventional and ecological Cabernet Sauvignon red wines were used. These wines were produced in 2018 in cultivation area Zmajevac, Baranja vineyard, Croatia. Conventional viticulture included 6 (in rainy seasons more) treatments of grapevine with commercial copper-based additives. Ecological viticulture included 10 treatments of grapevine with elementary sulphur and copper (up to 3 kg/ha in one vegetation until the flowering stage). Additional treatments of ecologically produced grapes included the application of herbal additives with EKO certificate, flavonoids, amino acids, or Neem oil. Sulphur dioxide was used minimally. 

### 2.3. Reverse Osmosis Process

The reverse osmosis process of red wine was conducted in a LabUnit M20 laboratory filter (De Danske Sukkerfabrikker, Nakskov, Denmark) equipped with a plate module and six flat sheet polyamide membranes. For wine concentration, Alfa Laval RO98pHt M20 membranes were used due to their characteristics: pH from 2 to 11, maximum pressure 5.5 MPa, maximum temperature 60 °C, and NaCl rejection above 98%; measured at 2000 ppm, 1.6 MPa, and 25 °C. The surface of one membrane was 0.0289 m^2^. During the concentration of conventional and ecological red wines, four different pressures (2.5, 3.5, 4.5, and 5.5 MPa) and two temperature regimes (with and without cooling) were applied. The initial wine volume of 3 L was separated into 1.3 L of retentate and 1.7 L of permeate. The initial retentate temperature of the wine was 15 °C, and it was measured every 4 min during the reverse osmosis process, along with the permeate volume. For better comparison with the initial wine, retentates were diluted with distilled water to the initial volume.

### 2.4. Determination of Processing Parameters

In order to calculate permeate flux (*J*), the following formula was used:

*J* = *V_p_*/(*A* × *t*) (1)

where *V_p_* is permeate volume (L), *A* is the surface of a membrane (m^2^), and *t* is the process duration (h). The volume reduction factor (*VRF*) was calculated according to the formula:

*VRF* = *V_f_*/*V_r_* (2)

where *V_f_* is the initial wine volume (L) and *V_r_* is the retentate volume (L). Water flux was measured before and after each experimental run, in order to calculate the fouling index (%):

*FI* = (1 − *J_W1_*/*J_W0_*) × 100 (3)

where *J_W0_* and *J_W1_* are the water fluxes (L/m^2^h) before and after wine concentration, respectively.

### 2.5. Determination of Phenolic Compounds

Phenolic compounds in initial conventional and ecological wine and their reverse osmosis retentates were determined spectrophotometrically, and they included: total polyphenols content determined by the Folin–Ciocalteu method [23], with results expressed as gallic acid equivalents (g GAE/L); total flavonoids content determined according to Kim et al. [24], with results expressed as catechin equivalents (g CE/L); and monomeric anthocyanins content (pH-differential method) and polymeric colour determined according to Giusti and Wrolstad [25]. Three repetitions were made for each sample.

### 2.6. Determination of Antioxidant Activity

Antioxidant activities in the analysed samples were determined spectrophotometrically according to four different assays: DPPH (2,2-diphenyl-1-picrylhydrazyl) [26], ABTS (2,20-azinobis3-ethylbenzothiazoline-6-sulfonic acid)) [27], FRAP (ferric-reducing/antioxidant power assay) [28], and CUPRAC (cupric-reducing antioxidant capacity) [29]. Results were expressed as Trolox equivalents (μmol TE/100 mL) and as the average value of three repetitions.

### 2.7. Determination of Individual Phenolic Compounds

Individual phenolic compounds were determined by a 1260 Infinity high-performance liquid chromatography (HPLC) system (Agilent Technologies, Santa Clara, CA, USA) equipped with a Poroshell 120 EC-C18 column (4.6 × 100 mm, 2.7 μm), quaternary pump, and diode array detector (DAD). As mobile phase A and B, 0.1% H_3_PO_4_ and 100% methanol were used, respectively. Two different methods were used: one for determination of individual phenolics, and other for determination of anthocyanins, according to Ivić et al. [30].

### 2.8. Determination of Colour Parameters

Colour parameters of CIELab system (L*, a*, b*, C*, and °h) in the initial wines and reverse osmosis retentates were determined by a CR-400 chromometer (Konica Minolta, Inc., Osaka, Japan). The lightness of a sample ranged from black (0) to white (100), and it was marked with L*. Parameter a* represented redness (+) or greenness (−), and parameter b* indicated yellowness (+) or blueness (−). Colour saturation was marked with C*, and °h indicated the hue angle [6,30,31]. Colour measurements for each sample were conducted in triplicates. In order to determine the colour difference between reverse osmosis retentates and corresponding initial wine, parameter ΔE* was calculated according to the formula:

ΔE* = [(ΔL*)^2^ + (Δa*)^2^ + (Δb*)^2^]^1/2^ (4)

### 2.9. Statistical Analysis

The results were analysed in the STATISTICA 13.1 (StatSoft Inc., Tulsa, OK, USA) software program, in which analysis of variance (ANOVA), post hoc Fisher’s least significant difference (LSD) test (*p* < 0.05), and principal component analysis (PCA) were conducted. The average values and standard deviations of repetitions were calculated. MS Excel (Microsoft Office Professional, 2016) was used for correlation coefficient calculation.

## 3. Results

### 3.1. Reverse Osmosis Process

The concentration process of conventional and ecological Cabernet Sauvignon red wine by RO was conducted at 2.5, 3.5, 4.5, and 5.5 MPa with and without cooling. During each experimental run, the permeate volume, retentate temperature, and process duration were measured. The same results were obtained during the reverse osmosis process of both wines. Table 1 presents the average permeate flux and final retentate temperature (FRT) obtained at different pressures and temperature regimes during the concentration of conventional and ecological red wines. The same results regarding permeate flux and retentate temperature were obtained for both wines, conventional and ecological.

It was observed that the pressure increase resulted in a higher average permeate flux in both temperature regimes. The highest permeate flux was obtained at 5.5 MPa with and without cooling (11.6 and 14.8 L/m^2^h, respectively). The lowest permeate flux was achieved at 2.5 MPa in both temperature regimes (3.4 L/m^2^h in the cooling regime and 5.4 L/m^2^h in the regime without cooling). When cooling was not applied, the permeate flux was 2.0 to 3.4 L/m^2^h higher than that obtained in the cooling regime at the same pressures. In addition, the regime without cooling resulted in a 13 to 16 °C higher FRT that increased with the pressure increment. Therefore, the highest FRT was measured for retentates obtained at 5.5 MPa without cooling (57.0 °C), and the lowest FRT in the retentates obtained at 2.5 MPa with cooling (36.0 °C).

**Figure 1:**
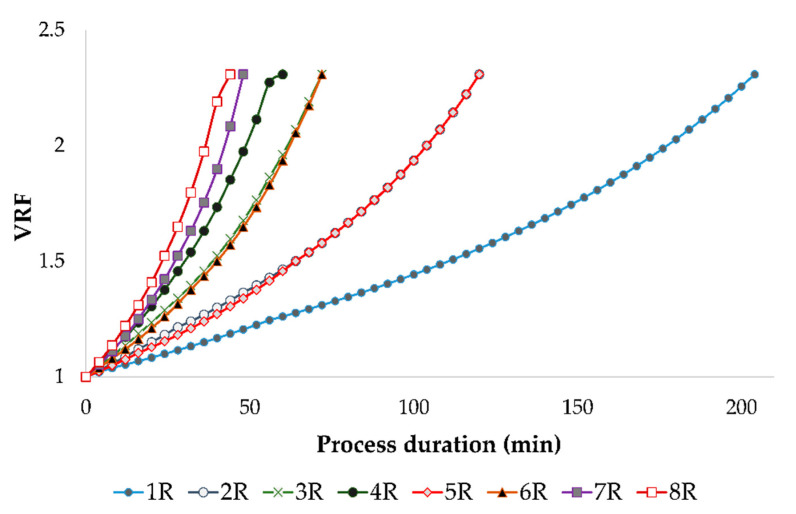
The change in volume reduction factor (VRF) value during reverse osmosis process of conventional and ecological red wines at different processing parameters. Abbreviations: R—reverse osmosis process; 1—2.5 MPa with cooling; 2—3.5 MPa with cooling; 3—4.5 MPa with cooling; 4—5.5 MPa with cooling; 5—2.5 MPa without cooling; 6—3.5 MPa without cooling; 7—4.5 MPa without cooling; 8—5.5 MPa without cooling.

**Figure 2:**
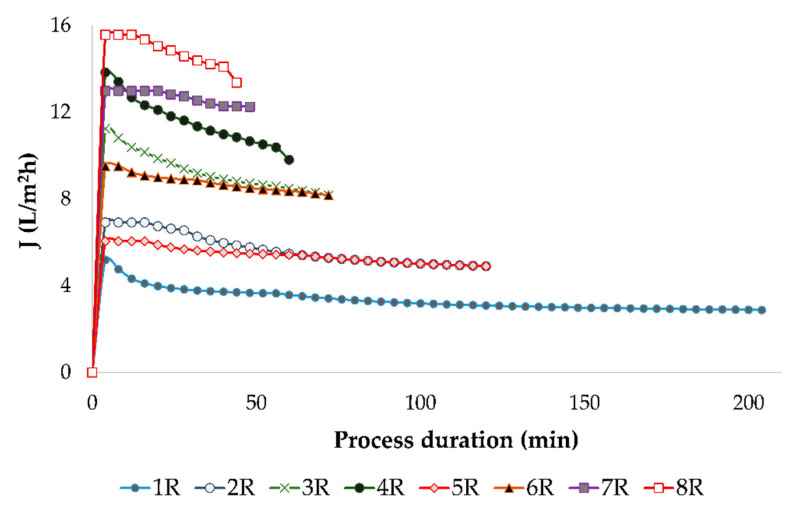
Permeate flux (*J*) decline during reverse osmosis process of conventional and ecological red wines at different processing parameters. Abbreviations: R—reverse osmosis process; 1—2.5 MPa with cooling; 2—3.5 MPa with cooling; 3—4.5 MPa with cooling; 4—5.5 MPa with cooling; 5—2.5 MPa without cooling; 6—3.5 MPa without cooling; 7—4.5 MPa without cooling; 8—5.5 MPa without cooling.

In this study, the reverse osmosis process of red wine resulted in 1.3 L of retentate and 1.7 L of permeate. According to these values, the volume reduction ratio was calculated, and a value of 2.31 was obtained for each experimental run. The VRF value increased during the reverse osmosis process of red wine (Figure 1) as a result of the reduction of the retentate volume. However, if the permeate flux was lower (at lower pressures and cooling regime), it took more time to obtain the desired VRF. Along with that, a permeate flux decline was observed in each experimental run, which is presented in Figure 2. The permeate flux decline was a result of membrane fouling, retention of small molecules, an osmotic pressure increase at the membrane surface, and concentration polarization [6]. Further, it also was observed that at 2.5 MPa with cooling, the reverse osmosis process lasted for 204 min. When a higher pressure or higher temperature were applied, the RO process was shorter, and the shortest one was at 5.5 MPa without cooling (44 min).

**Figure 3:**
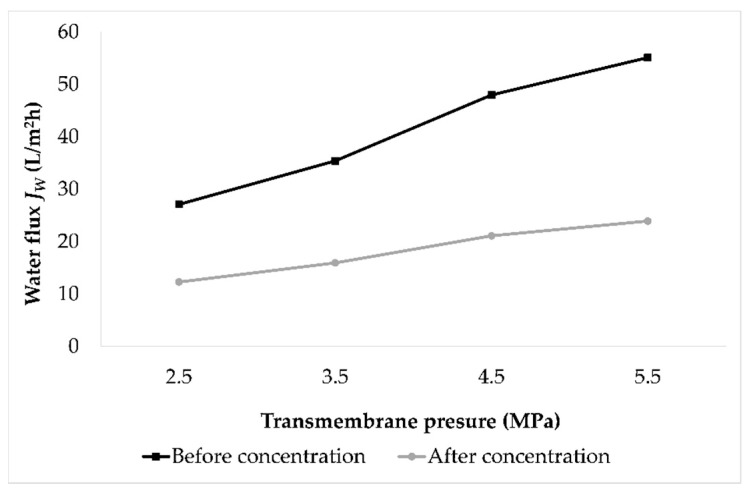
Water fluxes *J_W_* (L/m^2^h) before and after concentration of conventional and ecological Cabernet Sauvignon red wines by reverse osmosis measured at 2.5, 3.5, 4.5, and 5.5 MPa at 25 °C.

In order to estimate the membrane fouling that caused permeate flux decline, the fouling index was calculated. For that purpose, the pure water flux was measured at 2.5, 3.5, 4.5, and 5.5 MPa before and after each experimental run. Average values of fouling indices of all experiments at certain pressure were calculated, and are presented in Figure 3. It was observed that the water flux was higher at a higher pressure. However, after the concentration process, the water flux was significantly lower compared to the water flux before the concentration process at the same applied pressure. The water flux decrease was expressed as the fouling index (Table 2). The fouling index ranged from 54.68 to 56.61%, and was slightly higher when higher pressure was applied.

### 3.2. Retention of Phenolic Compounds

In the initial conventional and ecological red wines and their RO retentates obtained at 2.5, 3.5, 4.5, and 5.5 MPa with and without cooling, the total polyphenols content (TPC), total flavonoids content (TFC), monomeric anthocyanins content (MAC), and polymeric colour (PC) were determined. The results are presented in Table 3 and Table 4.

The results showed that the initial TPC value in the conventional (3.19 g/L) and ecological (3.34 g/L) wines decreased after reverse osmosis, and the total polyphenols content depended on the processing parameters. Pressure increase and retentate cooling resulted in higher retention of TPC than the opposite processing parameters, although the highest retention of TPC among ecological wine retentates (93.7%) was measured at 5.5 MPa with and without cooling. The highest retention of TPC in conventional wine retentates (90.3%) was obtained at 5.5 MPa with cooling. A pressure of 2.5 MPa resulted in the lowest retention of TPC in both wine retentates (around 73%), especially when cooling was not applied. 

Higher pressure was also favourable for TFC retention in both wine retentates. The highest retention of TFC among conventional wine retentates was obtained at 4.5 and 5.5 MPa with and without cooling (around 83%). On the other hand, the highest retention of TFC among ecological wine retentates was observed at 4.5 and 5.5 MPa with cooling (with no significant difference between the obtained values and initial value), and the regime without cooling resulted in a lower retention at same pressures. In the initial conventional and ecological wines, the TFC was 1.55 and 1.64 g/L, respectively. Among conventional wine retentates, the lowest retention of TFC was 71.6%, which was achieved at 2.5 MPa with and without cooling. In the ecological wine retentates, the lowest retention of TFC (74.4%) was achieved for retentates obtained at 2.5 and 3.5 MPa without cooling.

Lower temperatures and higher transmembrane pressures resulted in higher retention of MAC during reverse osmosis of conventional and ecological wines than the opposite operating conditions, although a significant loss was observed in both wine retentates. Initial conventional and ecological wines contained 151.41 and 103.83 mg/L, respectively, of monomeric anthocyanins. The highest loss of MAC, around 52% of initial concentration, was observed at 2.5 and 3.5 MPa without cooling in conventional wine retentates; and at 2.5 MPa without cooling in ecological wine retentates, 38.4% of the initial concentration was lost. On the other hand, at 5.5 MPa with cooling, the highest retention of MAC was achieved in both wine retentates (59.6% in conventional and 76.6% in ecological wine retentates). 

The polymeric colour in the initial conventional wine was 61.50%, and it increased to 62.15% in the cooling regime and 64.34% in the regime without cooling, with no significant difference among pressures. The PC in the initial ecological wine was 68.62%, and it did not significantly change after the RO process with cooling, but it increased after the RO process without cooling to 70.40%, with no significant difference among pressures.

Comparing the retention of TPC, TFC, and MAC between conventional and ecological red wines, it was observed that the retention was slightly higher in ecological wine retentates, especially at 5.5 MPa with cooling, where the highest retentions were achieved. Both initial wines contained similar total polyphenols and flavonoids content, and the initial conventional wine had a higher content of monomeric anthocyanins; however, after RO treatment, a higher loss of MAC occurred compared to the ecological wine.

### 3.3. Retention of Individual Phenolic Compounds

In the analysed samples, gallic acid, caffeic acid, caftaric acid, (+)-catechin, (−)-epicatechin, rutin, quercetin, quercetin derivative 1 and 2, and malvidin 3-glucoside and its derivative were determined, and the results are presented in Table 5 and Table 6.

The results showed that the initial conventional and ecological wines contained the same types of individual phenolic compounds, but in different concentrations. After the reverse osmosis process of both wines, a loss of individual phenolic compounds was observed, except for rutin in conventional wine retentates obtained at 3.5, 4.5, and 5.5 MPa with and without cooling, where concentrations were the same or higher than the initial concentration (0.95 mg/L). The highest concentration of rutin among conventional wine retentates was 1.20 mg/L, obtained at 5.5 MPa with cooling. 

During reverse osmosis treatment of conventional wine, higher pressure and retentate cooling resulted in higher retention of phenolic compounds compared to the opposite conditions. The initial concentrations of gallic acid, caffeic acid, caftaric acid, (+)-catechin, quercetin, and quercetin derivative 2 in conventional wine were 42.22, 2.71, 11.18, 88.71, 1.18, and 1.11 mg/L, respectively, and the highest retention of those compounds (above 90%) was obtained at 5.5 MPa with cooling. At same processing parameters, the highest retention of quercetin derivatives 1 was obtained (77.7% of initial concentration of 2.02 mg/L). Further, the highest retention of (−)-epicatechin (around 88% of initial concentration of 34.63 mg/L) was obtained at 3.5, 4.5, and 5.5 MPa with cooling, along with 4.5 and 5.5 MPa without cooling. The highest retention of malvidin 3-glucoside and its derivative were 95.5% and 92.4%, respectively, and it was obtained at 5.5 MPa at both temperature regimes for malvidin 3-glucoside, and at 5.5 MPa with cooling and 4.5 MPa without cooling for malvidin 3-glucoside derivative. The lowest retention of all compounds in conventional wine retentates was obtained at 2.5 MPa without cooling.

A similar trend was observed in the ecological wine retentates. The lowest retention of all compounds was obtained at 2.5 MPa without cooling, and the pressure increase and retentate cooling increased the retention of phenolic compounds. The highest retention of most compounds (gallic acid, caffeic acid, caftaric acid, (+)-catechin, and malvidin 3-glucoside and its derivative) was obtained at higher pressures (4.5 or 5.5 MPa or both) in the cooling regime, and the retention was above 90% of the initial concentration. The initial concentration of mentioned compounds in the ecological wine were 43.95, 2.10, 4.05, 42.18, 16.12, and 3.07 mg/L, respectively. The highest retention of (−)-epicatechin was around 75.2% at 4.5 and 5.5 MPa with cooling. Around 74% and 88% of the initial concentration of quercetin derivatives 1 and 2, respectively, was retained in the ecological wine retentates obtained at 3.5, 4.5, and 5.5 MPa with cooling. The same processing parameters, along with 3.5 MPa without cooling, resulted in the highest retention of rutin (around 86%). The concentration of quercetin in the initial ecological wine was 3.57 mg/L. While the highest retention of quercetin (68.1%) was obtained at 4.5 and 5.5 MPa with cooling, the regime without cooling resulted in a total loss of quercetin, except for 5.5 MPa, for which 0.39 mg/L of quercetin was found.

**Figure 4:**
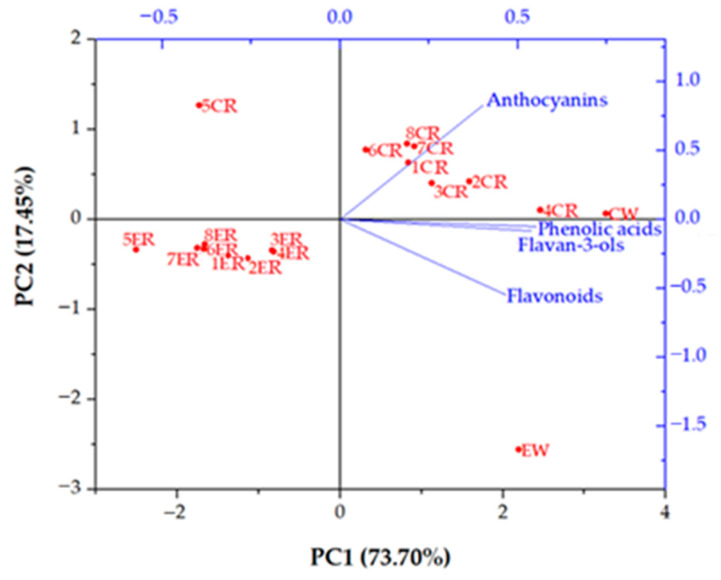
Principal component analysis (PCA) biplot of phenolic profiles of initial conventional and ecological red wines and their RO retentates. Abbreviations: CW—initial conventional wine; CR—reverse osmosis retentate of conventional wine; EW—initial ecological wine; ER—reverse osmosis retentate of ecological wine; 1—2.5 MPa with cooling; 2—3.5 MPa with cooling; 3—4.5 MPa with cooling; 4—5.5 MPa with cooling; 5—2.5 MPa without cooling; 6—3.5 MPa without cooling; 7—4.5 MPa without cooling; 8—5.5 MPa without cooling.

For a better comparison of the phenolic profile of initial conventional and ecological wine and their RO retentates, a principal component analysis (PCA) was conducted. For that purpose, all individual phenolic compounds were divided into four groups: phenolic acids (gallic, caffeic, and caftaric acid), flavan-3-ols (catechin and epicatechin), flavonoids (rutin, quercetin, and its two derivatives), and anthocyanins (malvidin 3-glucoside and its derivative). The PCA biplot (Figure 4) was divided by the principal component 1 (PC1), which accounted for 73.70% of the total variance; and principal component 2 (PC2), which accounted for 17.45% of the total variance. PC1 separated the samples according to the applied processing parameters. All ecological wine retentates were clustered on the negative side of PC1, along with the conventional wine retentates obtained at 2.5 MPa without cooling. The rest of the conventional wine retentates and initial wines were located on the positive side of PC1. PC2 separated the samples on the conventionally (positive side) and ecologically (negative side) produced wines and retentates. As mentioned, both initial wines contained the same types of individual phenolic compounds, but in different concentrations. This resulted in a phenolic profile of the initial conventional wine different from that of the ecological one, since they were located far away from each other on the PCA biplot. After the RO process, the ecological wine retentates were all located at the negative side of PC1 and PC2. They were far away from the initial ecological wine (positive side of PC1), meaning that the phenolic profile of the ecological wine significantly changed after RO treatment. The applied processing parameters influenced the retention of individual phenolic compounds, and there was a significant difference between the ecological wine retentates obtained at 2.5 MPa without cooling (5ER) and at 4.5 and 5.5 MPa with cooling (3ER and 4ER). The rest were clustered in the middle of them. Regarding the conventional wine retentates, they were located on the positive side of PC1 and PC2, the same as the initial conventional wine. The conventional wine retentate obtained at 2.5 MPa without cooling (5CR) was an exception, because it was located on the negative side of PC1, meaning that its phenolic profile was significantly different than the rest of the retentates and the initial conventional wine. At 5.5 MPa with cooling (4CR), the obtained retentate had the most similar phenolic profile as the initial conventional wine, and they were located near each other on the PCA biplot.

### 3.4. Antioxidant Activity 

In order to determine the antioxidant activity of the initial wine and RO retentates, four different methods were used: DPPH, ABTS, FRAP, and CUPRAC. The results are presented in Table 7 and Table 8.

The antioxidant activities obtained by the DPPH, ABTS, FRAP, and CUPRAC methods in the initial conventional (14.92, 35.18, 3.04, and 174.77 µmol/100 mL, respectively) and ecological (14.77, 33.46, 3.10, and 170.85 µmol/100 mL, respectively) Cabernet Sauvignon red wines were similar. In both wine retentates, a decrease in antioxidant activities was observed after the RO process. The decrease was influenced by processing parameters, and if higher pressure was applied, higher antioxidant activities were measured. The highest values of DPPH, ABTS, and CUPRAC among retentates were measured at 5.5 MPa with cooling. The regime without cooling resulted in lower antioxidant activities obtained by DPPH, ABTS, and CUPRAC than the cooling regime in both wine retentates, when comparing the same pressures. The highest antioxidant activities obtained by the FRAP method in both wine retentates were measured at 4.5 and 5.5 MPa with and without cooling, respectively. A pressure of 2.5 MPa in the regime without cooling resulted in the lowest antioxidant activities obtained by all four methods in both wine retentates. In these operating conditions, the antioxidant activities obtained by DPPH, ABTS, FRAP, and CUPRAC method were 4.26, 16.46, 2.11, and 112.65 µmol/100 mL in conventional wine retentates and 3.32, 22.30, 2.16, and 107.47 µmol/100 mL in ecological wine retentates, respectively, meaning that the antioxidant activity was 30 to 78% lower in both wine retentates than in the corresponding initial wine. The highest decrease in antioxidant activity at 2.5 MPa without cooling was measured by the DPPH method: 71.4% in conventional and 77.5% in ecological wine retentates.

It was observed that the decrease in antioxidant activities in both wine retentates followed a similar trend as the decrease in total polyphenols (TPC), flavonoids (TFC), and monomeric anthocyanin (MAC) content. To prove that, correlation coefficients (*r*) were calculated between TPC, TFC, MAC, and antioxidant activities obtained by DPPH, ABTS, FRAP, and CUPRAC. The results are presented in Table 9.

In Table 9, it can be observed that the correlation coefficient between TPC, TFC, MAC and DPPH, ABTS, FRAP, CUPRAC ranged from 0.454 to 0.959, indicating positive linear relationships. However, the closer the *r* value was to +1, the stronger the positive linear relationship was. Therefore, a strong positive linear relationship (*r* was above 0.7) was observed between TPC, TFC, MAC, and antioxidant activities obtained by DPPH, ABTS, and FRAP in both wine retentates. A strong positive relationship also was observed between TPC, TFC, MAC, and antioxidant activity obtained by CUPRAC in the ecological wine retentates. The *r* value between TPC and CUPRAC in conventional wine retentates was 0.710, indicating a still strong positive linear relationship. On the other hand, the *r* values between TFC, MAC, and CUPRAC in conventional wine retentates were 0.454 and 0.542, respectively, indicating a moderate positive linear relationship.

### 3.5. Colour Parameters Determination

In order to determine the colour change of conventional and ecological red wine during concentration by reverse osmosis, the following parameters were determined: L*, a*, b*, C*, and °h. The colour change was then calculated, and ΔE* was obtained. The results are presented in Table 10 and Table 11.

The results showed that the lightness (L* values) in the initial conventional and ecological wines was 19.70, and it slightly increased after the RO process. The cooling regime resulted in slightly lower L* values (19.91 in conventional and 20.30 in ecological wine retentates) than the regime without cooling (20.03 in conventional and 20.50 in ecological wine retentates), regardless of the pressure change. The a* value in the initial conventional wine was 1.98 and in the initial ecological wine was 2.15, and these values did not significantly change after the RO process when cooling was applied, but slightly decreased in the regime without cooling, with no significant difference among pressures. The b* values (1.14 in initial conventional and 1.07 in initial ecological wine) did not significantly change after the RO process at different processing parameters. The °h values decreased from 35.80 in the initial conventional wine to 30.67 in the conventional wine retentates obtained with cooling, and to 29.90 in the ones obtained without cooling, with no significant difference among pressures. The °h values in the ecological wine retentates also decreased compared to the initial wine (33.54), but there were differences among retentates regarding processing parameters. The highest °h values among ecological wine retentates were obtained at 2.5, 3.5, and 5.5 MPa with cooling. The regime without cooling resulted in slightly lower values of hue angle than the cooling regime. Further, the C* parameter in the initial conventional (1.94) and initial ecological (1.58) wines increased in both wine retentates. Pressure increase did not have a significant influence on C* value in conventional wine retentates, but the temperature increase in the regime without cooling resulted in slightly higher C* values than in the cooling regime. Among ecological wine retentates, the highest C* value was measured at 5.5 MPa with cooling (3.24). In order to determine the colour change, ΔE* was calculated, and the results showed that this value was lower than 0.40 in the conventional wine retentates and lower than 0.83 in the ecological wine retentates, depending on the applied temperature regime. The pressure change did not have a significant influence on the colour change in the conventional wine retentates, but higher temperatures in the regime without cooling resulted in slightly higher ΔE* values (around 0.35) than in the cooling regime (around 0.22). In the ecological wine retentates, the pressure did not significantly affect the ΔE* in the regime without cooling, but in the cooling regime, slight differences between pressures were observed, and the lowest ΔE* was obtained at 2.5 MPa with cooling (0.55).

## 4. Discussion

In this study, conventional and ecological Cabernet Sauvignon red wines were subjected to a reverse osmosis (RO) concentration process at pressures of 2.5, 3.5, 4.5, and 5.5 MPa with and without cooling, in order to estimate the influence of processing parameters on the retention of phenolic compounds. During the experimental runs, permeate flux, retentate temperature, and their volumes were monitored. Higher applied pressure resulted in higher permeate flux due to higher interaction of water with membrane hydrophilic parts, which increased water permeability through the membrane [30,32,33]. The higher the pressure, the higher the final retentate temperature, especially if cooling was not applied. Temperature increase also resulted in higher permeate flux due to lower viscosity of the wine at higher temperatures [6,34]. Gurak et al. [35] obtained similar results during RO treatment of grape juice. They established that the pressure increase (40 to 60 bar) and temperature increase (20 to 40 °C) led to higher permeate flux. In a previous study [36], a temperature-dependence model of the RO process was established, and a temperature correction factor was calculated that supported the above-mentioned statements.

Further, variable processing parameters during the RO process had a great influence on the membrane performance and retentate quality. The change in permeate concentration, concentration polarization, membrane fouling, and hydraulic resistance resulted in flux decline and higher retention of compounds, as stated in a study conducted by Dimitriou et al. [37]. Latorre et al. [38] stated that the increase in operating pressure in a desalination plant resulted in a constant salt passage and higher water flux, meaning that the obtained permeate contained lower salt concentrations.

However, during the RO process, the retentate volume decreased as more permeate was separated. Further, the retention of different compounds on the membrane’s surface led to membrane fouling, which resulted in higher retention of bioactive compounds at the beginning of the process, but also resulted in a permeate flux decline that limited the RO process and lowered its efficiency [33,39,40]. Membrane fouling includes several mechanisms: colloidal particulate deposition (colloidal fouling), adsorption of organic matters (organic fouling), precipitation of inorganic salts (inorganic scaling), and accumulation of microbial growth (biofouling) [16,40,41]. Understanding the fouling mechanism and modelling of the fouling during the RO process was the centre of interest in several previous studies [16,40,41,42,43]. Membrane fouling is usually described through the fouling index (FI), representing the percentage drop in the clean water permeability [44], silt density index (SDI; not very reliable), and modified fouling index (MFI) and combined fouling index (CFI). These can be used for predicting the fouling behaviour of an RO system [40,45,46]. In a previous study [47], it was stated that polyphenols, polysaccharides, or proteins that were retained on the membrane formed a pseudo-membrane on the surface, increasing the retention of bioactive compounds and decreasing the permeate flux. Membrane fouling represents one of the major problems in membrane filtration, due to the accumulation of different compounds on the membrane’s surface, and results in higher salt rejection and significant permeate flux decline [16]. This phenomenon contributes to the retention of the bioactive compounds, but at the end of the process, it will result in low permeate flux, low productivity, and membrane damage [6,40,48]. In addition, in several previous studies, during RO treatment of chokeberry juice [33], grape juice [35], beer [49], and red wine [6], similar results regarding permeate flux decline were obtained. In this study, the fouling index calculation showed that membrane fouling existed during red wine concentration. It was usually a result of residual sugars, polyphenols, and organic acids accumulating on the membrane’s surface. Each compound contributed to the membrane fouling, formation of the fouling layer, and permeate flux decline [50].

Furthermore, the retention of bioactive compounds depended on several factors, such as membrane characteristics and pore size, polarity of the membrane and compounds, interactions between compounds and membranes, membrane fouling, and processing parameters [6,9,30]. Low-molecular-weight (MW) molecules, such as water and ethanol, could pass through the membrane because they were smaller than the membrane pore size. The molecular weight cut-off of RO membranes is usually around 200 Da or lower, meaning that molecules with an MW above 200 g/mol will be retained on the membrane’s surface in a high percentage [51]. These molecules, such as polysaccharides, polyphenols, salts, or colloids, contributed to cake formation, membrane fouling, and retention of bioactive compounds, as mentioned. Diban et al. [52] stated that molecules with an MW higher than MWCO could also pass through the membrane due to its hydrophobic or hydrophilic character, which was attracted to the hydrophobic/hydrophilic part of the membrane, increasing the permeability of a molecule.

In this study, in the initial wines and RO retentates, gallic acid, caftaric acid, caffeic acid, (+)-catechin, (−)-epicatechin, rutin, quercetin and its derivatives, and malvidin 3-glucoside and its derivative were determined. These compounds are characteristic of Cabernet Sauvignon red wine [6,53,54]. The representative of anthocyanins was malvidin 3-glucoside, as it is the most abundant one in red wines [55]. The results of this study showed that conventional and ecological Cabernet Sauvignons had the same types of phenolic compounds, but their concentrations differed in the initial wines. After the RO process, the phenolic profile changed, as could be observed in the PCA biplot. Usually, higher pressure and lower temperatures favoured the retention of phenolic compounds, but processing parameters did not affect each compound the same way. The retention of each compound depended on several mentioned factors (chemical properties of the membrane and compounds, membrane fouling, concentration polarization, etc.), but also on the electrical charge of the membrane, as well as interactions between compounds that increased their stability [6,56]. Further, the retention of individual phenolic compounds differed between the conventional and ecological wines. For example, at 5.5 MPa with cooling, the retention of phenolic acids, (-)-epicatechin, flavonoids, and malvidin 3-glucoside was slightly higher in conventional wine retentates than in ecological ones. The phenolic profile of conventional wine retentates obtained at these processing parameters was the most similar to the phenolic profile of the initial conventional wine. The retention of (+)-catechin, quercetin derivative 2, and malvidin 3-glucoside derivative was slightly higher in the ecological wine retentate obtained at 5.5 MPa with cooling compared to the conventional wine retentate obtained at the same processing parameters.

Along with the initial wine matrix, the pH of the initial feed and retentate played a large role in bioactive compound retention [57]. The pH influenced dissociation of the membrane active layer and phenolic compounds. Each membrane had an isoelectric point at a certain pH, at which its surface had a neutral charge, and the highest permeability of compounds could be achieved [58,59]. Most polyamide membranes have an isoelectric point at pH around 4.0 that is near wine pH. The pH of conventional wine used in this study was 3.92, and the ecological one was 3.75. If the pH was lower than the isoelectric point of the membrane, it was expected that the membrane would have a positive charge, and if it was higher, the membrane would have a negative charge. Mnif et al. [60] stated that at a higher pH (around 12.0), the retention of phenolic compounds increased from 65 to almost 90%. On the other hand, they also stated that adsorption of phenolic compounds on the membrane surface could occur, due to hydrophobic interactions between phenolic compounds and parts of a membrane, leading to higher membrane fouling.

For a better understanding of membrane surface roughness, compound adsorptions, colloid deposition, and fouling, the Derjaguin–Landau–Verwey–Overbeek (DLVO) theory was developed. The DLVO theory assumes that the net interactions between colloids and surface is a sum of the van der Waals force (U_VDW_) and the electrostatic interaction force (U_EL_) [61], and therefore all particles exhibit attractive and repulsive interactions. In a previous study, according to the DLVO theory, the roughness of a membrane increased the interactions with the colloids due to a lower energy barrier between them, compared with a smooth membrane surface [62]. Pei et al. [63] stated that for polyphenol particles under electrostatic repulsion with a membrane, at a higher ionic strength of the solution, the U_EL_ between particles and membrane could be weakened. Therefore, polyphenol retention could be higher if membrane characteristics were manipulated with higher salt diffusion and followed by pectin fouling.

Further, in this study, total polyphenols (TPC), flavonoids (TFC), monomeric anthocyanins (MAC) content, polymeric colour, and antioxidant activity were determined. As the phenolic compounds are known as strong antioxidants, the correlation coefficient was calculated to determine the linear relationship between TPC, TFC, MAC, and antioxidant activity measured by DPPH, ABTS, FRAP, and CUPRAC methods. All four methods have different principles, and one is usually not enough for representing wine total antioxidant activity [64]. The results showed that the loss of total polyphenols, flavonoids, or monomeric anthocyanins resulted in a decrease in antioxidant activity. As the higher pressure and retentate cooling favoured the retention of total phenolic compounds, the highest antioxidant activities were measured at higher pressure, especially 5.5 MPa in the cooling regime.

The retention of MAC followed the above-mentioned trend: higher pressure and lower temperature resulted in higher retention. On the other hand, pressure change did not have a significant influence on polymeric colour, but the temperature increase in the regime without cooling resulted in a higher polymeric colour percentage. Polymeric colour includes polymerised materials that are formed during the conversion of anthocyanins into undesirable brown or colourless compounds [65]. Therefore, the polymeric colour increase could also be a result of anthocyanin degradation due to the temperature increase.

Phenolic compounds are also responsible for wine colour, thus CIELab colour parameters were determined. The results showed that slight changes occurred regarding lightness, the a* parameter, hue angle, and colour saturation, and the change depended on the processing parameters. However, in order to estimate the colour change between the RO retentates and the corresponding initial wine, the ΔE* value was determined. If this value was lower than 1, the colour change was not visible to the human eye [65]. Therefore, the RO process of conventional and ecological red wines did not visibly change the colour of the retentates, as the ΔE* values were all lower than 1.

## 5. Conclusions

The results of this study showed that the reverse osmosis process could be applied for the concentration of conventional and ecological Cabernet Sauvignon red wines, but optimal processing parameters should be established in order to achieve the highest retention of bioactive compounds. A pressure increase from 2.5 to 5.5 MPa resulted in about a 65 to 70% higher average permeate flux, a 15% higher final retentate temperature, and a 2% higher fouling index. The retention of total phenolic compounds was higher if a higher pressure and lower temperature were applied in both wine retentates, but the retention of individual phenolic compounds also depended on several other factors (initial wine matrix, membrane characteristics and fouling, chemical composition of the compounds and their interaction with the membrane, etc.). The highest retention of individual phenolic compounds was achieved at 5.5 MPa, especially when cooling was applied. In this operating condition, more than 90% of gallic acid, caffeic acid, caftaric acid, (+)-catechin, and malvidin 3-glucoside and its derivative was retained in both wine retentates, compared to the initial concentration. The lowest retention of all phenolic compounds was observed at 2.5 MPa with cooling. According to the PCA biplot, the RO process resulted in a significant change in the phenolic profile in the ecological wine retentates; while in the conventional ones, minimal changes occurred at 5.5 MPa with cooling. The decrease in antioxidant activities showed a positive linear relationship with the decrease in total polyphenols, flavonoids, and monomeric anthocyanins. The highest antioxidant activities were measured at 5.5 MPa with cooling in both wine retentates, compared to the corresponding initial wine. A slight colour change occurred after the RO process of conventional and ecological red wines, but the human eye was not able to distinguish it, according to the CIELab system.

## Figures and Tables

**Table 1:** Average permeate flux *J_A_* (L/m^2^h) and final retentate temperature (FRT) of RO retentates obtained by concentration of conventional and ecological red wine by reverse osmosis.

Sample	*J_A_* (L/m^2^h)	FRT (°C)
1R	3.4	36.0
2R	5.7	38.0
3R	9.3	39.0
4R	11.6	42.0
5R	5.4	49.0
6R	8.8	53.0
7R	12.7	55.0
8R	14.8	57.0

Abbreviations: R—reverse osmosis process; 1—2.5 MPa with cooling; 2—3.5 MPa with cooling; 3—4.5 MPa with cooling; 4—5.5 MPa with cooling; 5—2.5 MPa without cooling; 6—3.5 MPa without cooling; 7—4.5 MPa without cooling; 8—5.5 MPa without cooling.

**Table 2:** Fouling index (%) of reverse osmosis membranes at four different pressures.

Pressure (MPa)	Fouling Index (%)
2.5	54.68
3.5	54.95
4.5	56.05
5.5	56.61

**Table 3:** Phenolic compounds content of initial conventional Cabernet Sauvignon red wine and retentates obtained by reverse osmosis at 2.5, 3.5, 4.5, and 5.5 MPa with cooling and without cooling.

Sample	TPC (g GAE/L)	TFC (g CE/L)	MAC (mg CGE/L)	PC (%)
CW	3.19 ± 0.06 ^e^	1.55 ± 0.04 ^d^	151.41 ± 0.49 ^e^	61.50 ± 0.22 ^a^
1CR	2.36 ± 0.04 ^a^	1.11 ± 0.01 ^a^	79.39 ± 0.97 ^b^	62.14 ± 0.32 ^b^
2CR	2.57 ± 0.05 ^bc^	1.17 ± 0.03 ^b^	79.70 ± 0.85 ^b^	62.11 ± 0.42 ^b^
3CR	2.66 ± 0.09 ^c^	1.27 ± 0.04 ^c^	86.99 ± 0.65 ^c^	62.22 ± 0.33 ^b^
4CR	2.88 ± 0.08 ^d^	1.26 ± 0.04 ^c^	90.19 ± 0.72 ^d^	62.12 ± 0.45 ^b^
5CR	2.34 ± 0.03 ^a^	1.13 ± 0.03 ^ab^	72.35 ± 0.84 ^a^	64.20 ± 0.21 ^c^
6CR	2.48 ± 0.04 ^b^	1.22 ± 0.03 ^bc^	72.09 ± 0.43 ^a^	64.01 ± 0.27 ^c^
7CR	2.55 ± 0.03 ^bc^	1.32 ± 0.04 ^c^	79.50 ± 0.84 ^b^	64.28 ± 0.30 ^c^
8CR	2.83 ± 0.04 ^d^	1.30 ± 0.04 ^c^	80.14 ± 0.11 ^b^	64.87 ± 0.24 ^c^

Within column, different superscript letters (^a^, ^b^, ^c^, ^d^, ^e^) indicate significantly different values (*p* < 0.05; ANOVA; Fisher’s LSD test). Abbreviations: CW—initial conventional wine; CR—reverse osmosis retentate of conventional wine; 1—2.5 MPa with cooling; 2—3.5 MPa with cooling; 3—4.5 MPa with cooling; 4—5.5 MPa with cooling; 5—2.5 MPa without cooling; 6—3.5 MPa without cooling; 7—4.5 MPa without cooling; 8—5.5 MPa without cooling; TPC—total polyphenols content; TFC—total flavonoids content; MAC—monomeric anthocyanins content; PC—polymeric colour; GAE –gallic acid equivalent; CE—catechin equivalent; CGE—cyanidin 3-glucoside equivalent.

**Table 4:** Phenolic compounds content of initial ecological Cabernet Sauvignon red wine and retentates obtained by reverse osmosis at 2.5, 3.5, 4.5, and 5.5 MPa with cooling and without cooling.

Sample	TPC (g GAE/L)	TFC (g CE/L)	MAC (mg CGE/L)	PC (%)
EW	3.34 ± 0.06 ^e^	1.64 ± 0.02 ^c^	103.83 ± 0.72 ^f^	68.62 ± 0.97 ^a^
1ER	2.81 ± 0.05 ^b^	1.39 ± 0.01 ^b^	65.55 ± 0.67 ^b^	68.75 ± 0.21 ^a^
2ER	2.90 ± 0.05 ^bc^	1.43 ± 0.03 ^b^	67.88 ± 0.70 ^c^	68.49 ± 0.35 ^a^
3ER	2.96 ± 0.07 ^c^	1.60 ± 0.05 ^c^	76.09 ± 0.83 ^d^	68.10 ± 0.59 ^a^
4ER	3.13 ± 0.06 ^d^	1.53 ± 0.08 ^bc^	79.53 ± 0.88 ^e^	68.87 ± 0.12 ^a^
5ER	2.47 ± 0.10 ^a^	1.22 ± 0.02 ^a^	63.91 ± 0.70 ^a^	70.52 ± 0.41 ^b^
6ER	2.54 ± 0.04 ^a^	1.28 ± 0.04 ^a^	66.56 ± 0.69 ^bc^	70.45 ± 0.50 ^b^
7ER	3.01 ± 0.04 ^c^	1.44 ± 0.04 ^b^	66.35 ± 0.73 ^bc^	70.18 ± 0.33 ^b^
8ER	3.05 ± 0.08 ^cd^	1.39 ± 0.06 ^b^	77.30 ± 0.84 ^d^	70.44 ± 0.27 ^b^

Within column, different superscript letters (^a^, ^b^, ^c^, ^d^, ^e^, ^f^) indicate significantly different values (*p* < 0.05; ANOVA; Fisher’s LSD test). Abbreviations: EW—initial ecological wine; ER—reverse osmosis retentate of ecological wine; 1—2.5 MPa with cooling; 2—3.5 MPa with cooling; 3—4.5 MPa with cooling; 4—5.5 MPa with cooling; 5—2.5 MPa without cooling; 6—3.5 MPa without cooling; 7—4.5 MPa without cooling; 8—5.5 MPa without cooling; TPC—total polyphenols content; TFC—total flavonoids content; MAC—monomeric anthocyanins content; PC—polymeric colour; GAE –gallic acid equivalent; CE—catechin equivalent; CGE—cyanidin 3-glucoside equivalent.

**Table 5:** Concentration (mg/L) of individual phenolic compounds in the initial conventional Cabernet Sauvignon red wine and retentates obtained by reverse osmosis at 2.5, 3.5, 4.5, and 5.5 MPa with cooling and without cooling.

Sample	Gallic Acid	Caffeic Acid	Caftaric Acid	(+)-Catechin	(−)-Epicatechin	Rutin	Quercetin	DQ1	DQ2	Malvidin 3-Glucoside	DM3-g
**CW**	42.22 ± 0.65 ^g^	2.71 ± 0.01 ^f^	11.18 ± 0.11 ^e^	88.71 ± 0.60 ^g^	34.63 ± 0.16 ^d^	0.95 ± 0.02 ^b^	1.18 ± 0.01 ^f^	2.02 ± 0.04 ^g^	1.11 ± 0.01 ^e^	38.57 ± 0.01 ^g^	8.27 ± 0.01 ^e^
**1CR**	37.37 ± 0.18 ^d^	2.24 ± 0.01 ^b^	9.47 ± 0.02 ^c^	73.06 ± 0.80 ^d^	28.88 ± 0.14 ^b^	0.90 ± 0.09 ^b^	0.83 ± 0.01 ^d^	1.26 ± 0.02 ^c^	0.88 ± 0.01 ^b^	35.28 ± 0.05 ^b^	7.35 ± 0.08 ^ab^
**2CR**	38.96 ± 0.08 ^e^	2.39 ± 0.01 ^d^	9.94 ± 0.01 ^d^	76.70 ± 0.61 ^e^	30.53 ± 0.09 ^c^	1.07 ± 0.02 ^c^	1.19 ± 0.01 ^f^	1.25 ± 0.01 ^c^	0.92 ± 0.01 ^c^	36.23 ± 0.07 ^d^	7.41 ± 0.03 ^b^
**3CR**	37.55 ± 0.25 ^d^	2.34 ± 0.01 ^c^	9.92 ± 0.12 ^d^	70.14 ± 0.25 ^c^	30.33 ± 0.36 ^c^	1.17 ± 0.01 ^d^	0.92 ± 0.04 ^e^	1.48 ± 0.02 ^e^	0.96 ± 0.03 ^c^	36.35 ± 0.05 ^d^	7.36 ± 0.06 ^ab^
**4CR**	41.34 ± 0.05 ^f^	2.53 ± 0.01 ^e^	9.98 ± 0.03 ^d^	82.95 ± 0.69 ^f^	30.60 ± 0.10 ^c^	1.20 ± 0.01 ^e^	1.18 ± 0.01 ^f^	1.57 ± 0.01 ^f^	1.02 ± 0.01 ^d^	36.65 ± 0.11 ^ef^	7.64 ± 0.03 ^d^
**5CR**	28.99 ± 0.11 ^a^	1.73 ± 0.01 ^a^	7.08 ± 0.03 ^a^	53.50 ± 0.28 ^a^	27.36 ± 0.23 ^a^	0.85 ± 0.01 ^a^	0.49 ± 0.01 ^a^	1.00 ± 0.01 ^a^	0.68 ± 0.01 ^a^	34.99 ± 0.03 ^a^	7.26 ± 0.04 ^a^
**6CR**	34.45 ± 0.11 ^b^	2.35 ± 0.04 ^cd^	8.87 ± 0.01 ^b^	66.25 ± 0.05 ^b^	32.51 ± 0.04 ^b^	1.05 ± 0.01 ^c^	0.60 ± 0.03 ^c^	1.32 ± 0.01 ^d^	0.85 ± 0.01 ^b^	36.00 ± 0.02 ^c^	7.40 ± 0.07 ^bc^
**7CR**	37.16 ± 0.19 ^d^	2.28 ± 0.05 ^bc^	9.52 ± 0.11 ^c^	74.20 ± 0.49 ^d^	30.23 ± 0.15 ^c^	0.99 ± 0.02 ^b^	0.66 ± 0.02 ^c^	1.16 ± 0.02 ^b^	0.85 ± 0.03 ^b^	36.66 ± 0.01 ^e^	7.60 ± 0.09 ^cd^
**8CR**	36.33 ± 0.09 ^c^	2.25 ± 0.02 ^b^	10.07 ± 0.19 ^d^	74.38 ± 0.43 ^d^	30.62 ± 0.15 ^c^	1.03 ± 0.06 ^b^	0.54 ± 0.02 ^b^	1.21 ± 0.03 ^c^	0.86 ± 0.01 ^b^	36.84 ± 0.08 ^f^	7.50 ± 0.03 ^c^

Significant differences (*p* < 0.05) between samples are indicated by different superscript letters within the column (ANOVA; Fisher’s LSD test). Abbreviations: CW—initial conventional wine; CR—reverse osmosis retentate of conventional wine; 1—2.5 MPa with cooling; 2—3.5 MPa with cooling; 3—4.5 MPa with cooling; 4—5.5 MPa with cooling; 5—2.5 MPa without cooling; 6—3.5 MPa without cooling; 7—4.5 MPa without cooling; 8—5.5 MPa without cooling; DQ1 and DQ2—quercetin derivative 1 and quercetin derivative 2; DM3-g—malvidin 3-glucoside derivative.

**Table 6:** Concentration (mg/L) of individual phenolic compounds in the initial ecological Cabernet Sauvignon red wine and retentates obtained by reverse osmosis at 2.5, 3.5, 4.5, and 5.5 MPa with cooling and without cooling.

Sample	Gallic Acid	Caffeic Acid	Caftaric Acid	(+)-Catechin	(−)-Epicatechin	Rutin	Quercetin	DQ1	DQ2	Malvidin 3-Glucoside	DM3-g
**EW**	43.95 ± 0.60 ^f^	2.10 ± 0.01 ^e^	4.05 ± 0.01 ^f^	42.18 ± 0.34 ^g^	69.80 ± 1.61 ^e^	1.59 ± 0.01 ^e^	3.57 ± 0.04 ^e^	1.43 ± 0.01 ^e^	1.20 ± 0.01 ^d^	16.12 ± 0.10 ^g^	3.07 ± 0.01 ^d^
**1ER**	39.60 ± 0.10 ^c^	1.78 ± 0.01 ^b^	3.59 ± 0.01 ^d^	38.61 ± 0.14 ^d^	49.40 ± 0.07 ^b^	1.30 ± 0.01 ^c^	1.63 ± 0.03 ^b^	0.80 ± 0.01 ^a^	0.98 ± 0.03 ^b^	14.42 ± 0.09 ^c^	2.43 ± 0.01 ^b^
**2ER**	40.65 ± 0.02 ^d^	1.83 ± 0.03 ^bc^	3.75 ± 0.05 ^e^	39.89 ± 0.03 ^e^	50.37 ± 0.49 ^c^	1.33 ± 0.04 ^cd^	2.07 ± 0.01 ^c^	1.06 ± 0.06 ^d^	1.06 ± 0.01 ^c^	14.21 ± 0.03 ^b^	2.41 ± 0.03 ^c^
**3ER**	41.86 ± 0.31 ^e^	1.90 ± 0.01 ^d^	3.74 ± 0.02 ^e^	39.94 ± 0.15 ^e^	52.49 ± 0.08 ^d^	1.38 ± 0.04 ^d^	2.42 ± 0.01 ^d^	1.02 ± 0.01 ^d^	1.04 ± 0.05 ^c^	15.53 ± 0.09 ^g^	3.08 ± 0.03 ^d^
**4ER**	41.45 ± 0.23 ^e^	1.93 ± 0.03 ^d^	3.79 ± 0.01 ^e^	41.29 ± 0.12 ^f^	52.44 ± 0.10 ^d^	1.34 ± 0.01 ^d^	2.43 ± 0.01 ^d^	1.01 ± 0.03 ^d^	1.02 ± 0.04 ^c^	15.26 ± 0.01 ^f^	3.12 ± 0.04 ^d^
**5ER**	34.29 ± 0.57 ^a^	1.56 ± 0.03 ^a^	3.06 ± 0.03 ^a^	33.07 ± 0.95 ^a^	46.15 ± 0.06 ^a^	1.14 ± 0.02 ^a^	-	0.83 ± 0.01 ^a^	0.84 ± 0.01 ^a^	13.08 ± 0.12 ^a^	2.21 ± 0.02 ^a^
**6ER**	37.28 ± 0.67 ^b^	1.87 ± 0.01 ^c^	3.28 ± 0.02 ^b^	36.31 ± 0.10 ^c^	50.70 ± 0.40 ^c^	1.35 ± 0.04 ^cd^	-	0.93 ± 0.01 ^b^	0.98 ± 0.01 ^b^	14.62 ± 0.09 ^d^	2.46 ± 0.01 ^c^
**7ER**	37.11 ± 0.29 ^b^	1.80 ± 0.07 ^bc^	3.45 ± 0.05 ^c^	35.03 ± 0.08 ^b^	50.93 ± 0.51 ^c^	1.32 ± 0.01 ^c^	-	0.98 ± 0.01 ^c^	0.99 ± 0.01 ^b^	14.60 ± 0.03 ^d^	2.48 ± 0.01 ^c^
**8ER**	37.35 ± 0.52 ^b^	1.77 ± 0.03 ^b^	3.48 ± 0.02 ^c^	35.99 ± 0.57 ^c^	50.87 ± 0.43 ^c^	1.26 ± 0.01 ^b^	0.39 ± 0.01 ^a^	0.97 ± 0.01 ^c^	0.93 ± 0.05 ^b^	14.79 ± 0.01 ^e^	3.10 ± 0.06 ^d^

Significant differences (*p* < 0.05) between samples are indicated by different superscript letters within the column (ANOVA; Fisher’s LSD test). Abbreviations: EW—initial ecological wine; ER—reverse osmosis retentate of ecological wine; 1—2.5 MPa with cooling; 2—3.5 MPa with cooling; 3—4.5 MPa with cooling; 4—5.5 MPa with cooling; 5—2.5 MPa without cooling; 6—3.5 MPa without cooling; 7—4.5 MPa without cooling; 8—5.5 MPa without cooling. DQ1 and DQ2—quercetin derivative 1 and quercetin derivative 2; DM3-g—malvidin 3-glucoside derivative.

**Table 7:** Antioxidant activity determined by DPPH, ABTS, FRAP, and CUPRAC in initial conventional Cabernet Sauvignon wine and retentates obtained by reverse osmosis at 2.5, 3.5, 4.5, and 5.5 MPa with cooling and without cooling.

Sample	DPPH (µmol TE/100 mL)	ABTS (µmol TE/100 mL)	FRAP (µmol TE/100 mL)	CUPRAC (µmol TE/100 mL)
CW	14.92 ± 0.97 ^f^	35.18 ± 0.15 ^h^	3.04 ± 0.15 ^e^	174.77 ± 1.07 ^f^
1CR	5.23 ± 0.42 ^b^	20.47 ± 0.19 ^c^	2.37 ± 0.03 ^b^	159.56 ± 1.94 ^c^
2CR	7.49 ± 0.41 ^c^	25.14 ± 0.18 ^e^	2.48 ± 0.03 ^c^	164.34 ± 1.04 ^d^
3CR	10.10 ± 0.13 ^d^	25.80 ± 0.48 ^e^	2.60 ± 0.03 ^d^	166.27 ± 2.16 ^d^
4CR	12.40 ± 0.38 ^e^	31.20 ± 0.06 ^g^	2.65 ± 0.02 ^d^	171.20 ± 1.49 ^e^
5CR	4.26 ± 0.46 ^a^	16.46 ± 0.24 ^a^	2.11 ± 0.08 ^a^	112.65 ± 2.01 ^a^
6CR	5.47 ± 0.42 ^b^	18.93 ± 0.17 ^b^	2.23 ± 0.04 ^a^	134.94 ± 2.68 ^b^
7CR	7.14 ± 0.27 ^c^	21.37 ± 0.16 ^d^	2.52 ± 0.05 ^cd^	137.65 ± 2.91 ^b^
8CR	7.37 ± 0.27 ^c^	27.01 ± 0.32 ^f^	2.56 ± 0.05 ^cd^	164.88 ± 2.32 ^d^

Within column, different superscript letters indicate significant differences among samples (*p* < 0.05; ANOVA; Fisher’s LSD test). Abbreviations: CW—initial conventional wine; CR—reverse osmosis retentate of conventional wine; 1—2.5 MPa with cooling; 2—3.5 MPa with cooling; 3—4.5 MPa with cooling; 4—5.5 MPa with cooling; 5—2.5 MPa without cooling; 6—3.5 MPa without cooling; 7—4.5 MPa without cooling; 8—5.5 MPa without cooling; TE—Trolox equivalent.

**Table 8:** Antioxidant activity determined by DPPH, ABTS, FRAP, and CUPRAC in initial ecological Cabernet Sauvignon wine and retentates obtained by reverse osmosis at 2.5, 3.5, 4.5, and 5.5 MPa with cooling and without cooling.

Sample	DPPH (µmol TE/100 mL)	ABTS (µmol TE/100 mL)	FRAP (µmol TE/100 mL)	CUPRAC (µmol TE/100 mL)
EW	14.77 ± 0.72 ^g^	33.46 ± 0.59 ^f^	3.10 ± 0.13 ^e^	170.85 ± 1.53 ^g^
1ER	6.12 ± 0.66 ^cd^	27.76 ± 0.11 ^b^	2.38 ± 0.07 ^b^	128.08 ± 1.47 ^d^
2ER	7.16 ± 0.65 ^d^	28.54 ± 0.12 ^c^	2.49 ± 0.08 ^c^	139.88 ± 0.18 ^e^
3ER	10.85 ± 0.37 ^e^	32.09 ± 0.28 ^e^	2.61 ± 0.07 ^cd^	138.75 ± 1.28 ^e^
4ER	13.16 ± 0.35 ^f^	32.18 ± 0.41 ^e^	2.66 ± 0.08 ^d^	161.47 ± 1.85 ^f^
5ER	3.32 ± 0.41 ^a^	22.30 ± 0.11 ^a^	2.16 ± 0.06 ^a^	107.47 ± 2.10 ^a^
6ER	4.58 ± 0.40 ^b^	27.25 ± 0.22 ^b^	2.24 ± 0.02 ^a^	115.07 ± 0.40 ^b^
7ER	5.73 ± 0.33 ^c^	27.80 ± 0.12 ^b^	2.55 ± 0.04 ^cd^	123.05 ± 1.67 ^c^
8ER	5.05 ± 0.36 ^bc^	30.15 ± 0.19 ^d^	2.60 ± 0.02 ^cd^	140.10 ± 2.22 ^e^

Within column, different superscript letters indicate significant differences among samples (*p* < 0.05; ANOVA; Fisher’s LSD test). Abbreviations: EW—initial ecological wine; ER—reverse osmosis retentate of ecological wine; 1—2.5 MPa with cooling; 2—3.5 MPa with cooling; 3—4.5 MPa with cooling; 4—5.5 MPa with cooling; 5—2.5 MPa without cooling; 6—3.5 MPa without cooling; 7—4.5 MPa without cooling; 8—5.5 MPa without cooling; TE—Trolox equivalent.

**Table 9:** Correlation coefficients (*r*) between TPC, TFC, MAC, and antioxidant activities obtained by DPPH, ABTS, FRAP, and CUPRAC methods in conventional and ecological Cabernet Sauvignon wines and their RO retentates at 2.5, 3.5, 4.5, and 5.5 MPa, with cooling and without cooling.

	Conventional Wine			Ecological Wine		
	TPC	TFC	MAC	TPC	TFC	MAC
DPPH	0.924	0.806	0.842	0.790	0.913	0.825
ABTS	0.959	0.764	0.799	0.874	0.923	0.776
FRAP	0.927	0.869	0.871	0.953	0.893	0.941
CUPRAC	0.710	0.454	0.542	0.905	0.859	0.859

**Table 10:** Colour parameters (L*, a*, b*, °h, and C*) in initial conventional Cabernet Sauvignon red wine and retentates obtained by reverse osmosis at 2.5, 3.5, 4.5, and 5.5 MPa with cooling and without cooling.

Sample	L*	a*	b*	°h	C*	ΔE*
CW	19.70 ± 0.01 ^a^	1.98 ± 0.03 ^b^	1.14 ± 0.03 ^a^	35.80 ± 0.64 ^c^	1.94 ± 0.02 ^a^	-
1CR	19.91 ± 0.01 ^b^	1.99 ± 0.05 ^b^	1.17 ± 0.02 ^a^	30.75± 0.61 ^b^	2.32 ± 0.04 ^b^	0.22 ± 0.01 ^a^
2CR	19.92 ± 0.02 ^b^	2.03 ± 0.02 ^b^	1.13 ± 0.02 ^a^	30.47± 0.35 ^b^	2.33 ± 0.05 ^b^	0.23 ± 0.01 ^a^
3CR	19.91 ± 0.01 ^b^	1.99 ± 0.03 ^b^	1.14 ± 0.01 ^a^	30.75± 0.29 ^b^	2.38 ± 0.03 ^b^	0.21 ± 0.02 ^a^
4CR	19.91 ± 0.01 ^b^	2.06 ± 0.05 ^b^	1.15 ± 0.02 ^a^	30.71± 0.25 ^b^	2.39 ± 0.04 ^b^	0.22 ± 0.01 ^a^
5CR	20.04 ± 0.01 ^c^	1.86 ± 0.01 ^a^	1.16 ± 0.03 ^a^	29.52± 0.18 ^a^	2.52 ± 0.02 ^c^	0.36 ± 0.01 ^b^
6CR	20.07 ± 0.01 ^c^	1.86 ± 0.01 ^a^	1.13 ± 0.02 ^a^	29.33± 0.26 ^a^	2.48 ± 0.02 ^c^	0.39 ± 0.03 ^b^
7CR	20.00 ± 0.01 ^c^	1.86 ± 0.01 ^a^	1.17 ± 0.03 ^a^	30.24± 0.12 ^b^	2.57 ± 0.05 ^c^	0.32 ± 0.04 ^b^
8CR	20.01 ± 0.01 ^c^	1.87 ± 0.02 ^a^	1.12 ± 0.04 ^a^	30.51± 0.31 ^b^	2.63 ± 0.05 ^c^	0.33 ± 0.03 ^b^

Significant differences (*p* < 0.05) between samples are indicated by different superscript letters within the column (ANOVA; Fisher’s LSD test). Abbreviations: CW—initial conventional wine; CR—reverse osmosis retentate of conventional wine; 1—2.5 MPa with cooling; 2—3.5 MPa with cooling; 3—4.5 MPa with cooling; 4—5.5 MPa with cooling; 5—2.5 MPa without cooling; 6—3.5 MPa without cooling; 7—4.5 MPa without cooling; 8—5.5 MPa without cooling.

**Table 11:** Colour parameters (L*, a*, b*, °h, and C*) in initial ecological Cabernet Sauvignon red wine and retentates obtained by reverse osmosis at 2.5, 3.5, 4.5, and 5.5 MPa with cooling and without cooling.

Sample	L*	a*	b*	°h	C*	ΔE*
EW	19.70 ± 0.01 ^a^	2.15 ± 0.02 ^b^	1.07 ± 0.01 ^a^	33.54 ± 0.32 ^d^	1.58 ± 0.03 ^a^	-
1ER	20.25 ± 0.05 ^b^	2.13 ± 0.02 ^b^	1.08 ± 0.03 ^a^	30.16 ± 0.28 ^c^	2.74 ± 0.02 ^e^	0.55 ± 0.01 ^a^
2ER	20.30 ± 0.01 ^b^	2.16 ± 0.01 ^b^	1.04 ± 0.03 ^a^	30.51 ± 0.52 ^c^	2.50 ± 0.01 ^b^	0.60 ± 0.01 ^b^
3ER	20.35 ± 0.03 ^b^	2.16 ± 0.01 ^b^	1.04 ± 0.01 ^a^	28.79 ± 0.28 ^a^	2.57 ± 0.01 ^c^	0.65 ± 0.04 ^c^
4ER	20.31 ± 0.01 ^b^	2.12 ± 0.03 ^b^	1.05 ± 0.01 ^a^	30.63 ± 0.21 ^c^	3.24 ± 0.02 ^f^	0.62 ± 0.02 ^bc^
5ER	20.51 ± 0.02 ^c^	2.04 ± 0.02 ^a^	1.06 ± 0.01 ^a^	29.16 ± 0.19 ^a^	2.80 ± 0.03 ^e^	0.82 ± 0.01 ^d^
6ER	20.49 ± 0.01 ^c^	2.06 ± 0.01 ^a^	1.04 ± 0.03 ^a^	29.75 ± 0.01 ^b^	2.64 ± 0.02 ^d^	0.80 ± 0.02 ^d^
7ER	20.49 ± 0.02 ^c^	2.02 ± 0.03 ^a^	1.02 ± 0.01 ^a^	29.15 ± 0.48 ^a^	2.50 ± 0.02 ^b^	0.80 ± 0.02 ^d^
8ER	20.49 ± 0.02 ^c^	2.04 ± 0.01 ^a^	1.05 ± 0.02 ^a^	28.91 ± 0.93 ^a^	2.83 ± 0.04 ^e^	0.79 ± 0.03 ^d^

Significant differences (*p* < 0.05) between samples are indicated by different superscript letters within the column (ANOVA; Fisher’s LSD test). Abbreviations: EW—initial ecological wine; ER—reverse osmosis retentate of ecological wine; 1—2.5 MPa with cooling; 2—3.5 MPa with cooling; 3—4.5 MPa with cooling; 4—5.5 MPa with cooling; 5—2.5 MPa without cooling; 6—3.5 MPa without cooling; 7—4.5 MPa without cooling; 8—5.5 MPa without cooling.

## Data Availability

Not available.

## Nomenclature

RO	Reverse osmosis
Da	Daltons (equalised with g/mol)
DPPH	2,2-diphenyl-1-picrylhydrazil
ABTS	2,2-azinobis(3-ethylbenzothiazoline sulfonic acid)
FRAP	Ferric-reducing/antioxidant power assay
CUPRAC	Cupric-reducing antioxidant capacity
TPC	Total polyphenols content
TFC	Total flavonoids content
MAC	Monomeric anthocyanins content
PC	Polymeric colour
GAE	Gallic acid equivalent
CE	Catechin equivalent
TE	Trolox equivalent
*r*	Correlation coefficient
HPLC	High-performance liquid chromatography
DAD	Diode array detector
L*	CIELab parameter – lightness
a*	CIELab parameter – redness/greenness
b*	CIELab parameter – yellowness/blueness
C*	CIELab parameter – colour saturation
°h	CIELab parameter – hue angle
ΔE*	CIELab parameter – colour difference
ANOVA	Analysis of variance
LSD	Fisher’s least significant difference test
PCA	Principal component analysis
PC1	Principal component 1
PC2	Principal component 2
*J*	Permeate flux (L/m^2^h)
*J_A_*	Average permeate flux (L/m^2^h)
*V_f_*	Initial wine volume (L)
*V_r_*	Retentate volume (L)
*V_p_*	Permeate volume (L)
*A*	Membrane surface (m^2^)
*t*	Process duration (h)
FI	Fouling index (%)
J_W0_	Clean water flux before wine concentration (L/m^2^h)
J_W1_	Clean water flux after wine concentration (L/m^2^h)
FRT	Final retentate temperature (°C)
CW	Conventional wine
CR	Reverse osmosis retentate of conventional wine
EW	Ecological wine
ER	Reverse osmosis retentate of ecological wine
VRF	Volume reduction factor
DQ1	Quercetin derivative 1
DQ2	Quercetin derivative 2
DM3-g	Malvidin 3-glucoside derivative
SDI	Silt density index
MFI	Modified fouling index
DLVO	Derjaguin–Landau–Verwey–Overbeek
